# Low expression of *Talin1* is associated with advanced pathological features in colorectal cancer patients

**DOI:** 10.1038/s41598-020-74810-6

**Published:** 2020-10-20

**Authors:** Somayeh Vafaei, Leili Saeednejad Zanjani, Zohreh Habibi Shams, Marzieh Naseri, Fahimeh Fattahi, Elmira Gheytanchi, Mahdi Alemrajabi, Marzieh Ebrahimi, Zahra Madjd

**Affiliations:** 1 Oncopathology Research Center, Iran University of Medical Sciences (IUMS), Hemmat Street (Highway), Next To Milad Tower, Tehran, Iran; 2 Department of Molecular Medicine, Faculty of Advanced Technologies in Medicine, Iran University of Medical Sciences, Tehran, Iran; 3 Student Research Committee, Iran University of Medical Sciences, Tehran, Iran; 4 Department of Stem Cells and Developmental Biology, Cell Science Research Center, Royan Institute for Stem Cell Biology and Technology, ACECR, 16635-148 Tehran, Iran; 5 Department of Pathology, Iran University of Medical Sciences, Tehran, Iran; 6 Firoozgar Clinical Research Development Center, Firoozgar Hospital (FCRDC), Iran University of Medical Sciences (IUMS), Tehran, Iran

**Keywords:** Cancer, Biomarkers, Oncology

## Abstract

To explore the proper prognostic markers for the likelihood of metastasis in CRC patients. Seventy-seven fresh CRC samples were collected to evaluate the mRNA level of the selected marker using Real-time PCR. Moreover, 648 formalin-fixed paraffin-embedded CRC tissues were gathered to evaluate protein expression by immunohistochemistry (IHC) on tissue microarrays. The results of Real-Time PCR showed that low expression of *Talin1* was significantly associated with advanced TNM stage (*p* = *0.034*) as well as gender (*p* = *0.029*) in mRNA levels. Similarly, IHC results indicated that a low level of cytoplasmic expression of *Talin1* was significantly associated with advanced TNM stage (*p* = *0.028*) as well as gender (*p* = *0.009*) in CRC patients. Moreover, decreased expression of cytoplasmic *Talin1* protein was found to be a significant predictor of worse disease-specific survival (DSS) (*p* = 0.011) in the univariate analysis. In addition, a significant difference was achieved (*p* = 0.039) in 5-year survival rates of DSS: 65% for low, 72% for moderate, and 88% for high *Talin1* protein expression. Observations showed that lower expression of *Talin1* at both the gene and protein level may drive the disparity of CRC patients’ outcomes via worse DSS and provide new insights into the development of progression indicators because of its correlation with increased tumor aggressiveness.

## Introduction

Colorectal cancer (CRC) is one of the frequent forms of solid tumor diagnosed in both sexes worldwide^1,2^. It is ranked as third in incidence but second in mortality in different countries, and most of deaths are caused by metastasis; hence, the prevention of subsequent metastasis is the main focus of clinical research^3,4^. Robust determinants of CRC prognosis include local involvement, lymphovascular invasion, positive surgical margin, regional lymph node metastasis, pre-operative elevation of CEA, and high tumor grade^5,6^. Moreover, traditional biomarkers, colono/sigmoidoscopy, imaging techniques (magnetic resonance imaging (MRI) and computed tomography (CT)) play specific roles as the gold standard in colorectal screening; however, they are not satisfactory enough^7^. Unfortunately, the early spread of tumor cells is usually not detectable, and access to more detailed information related to the identification of metastasis-specific molecular markers is urgently warranted.

Histopathological analysis as a prevalent standard test is applied to assess novel molecular biomarkers through simultaneous analysis of hundreds of tissue samples in the clinical setting^8^. The cancer patient's blood contains diverse tumor-derived materials, including circulating tumor cells (CTCs), and exosomes enable the minimally invasive monitoring of tumor evolution over time in the clinic^9,10^. It seems that achieving the molecular signature based on CTCs and exosomes, which is completely representative of the tumor, plays a significant role in precise theranostics and can be utilized in combination with clinicopathological staging. From a prognostic perspective, the identification of tumor metastasis-specific molecular markers which cover cancer heterogeneity and increase longitudinal surveillance of patients is essential for precision medicine^11^.

Although large‐scale studies have been carried out to identify novel biomarkers with great prognostic value, investigations of metastasis-related molecular markers should be increased to actualize the exact prediction of the time of metastasis. Advances in high throughput technology in omics data, especially transcriptomic approaches such as microarrays and RNA sequencing, would increase our knowledge of the molecular markers involved in CRC metastasis, resulting in the identification of potential therapeutic targets for CRC patients^12,13^. To the best of our knowledge, just a couple of studies have investigated the direct correlation between molecular metastasis cascades in CTCs and exosomes. Hence in the present study, databases were screened in order to analyze the common expression profile of these biomarkers as an essential step in understanding the mechanism of metastasis and in discovering the prognostic markers for CRC^14^. This study was performed using integrated analysis of bioinformatics data related to the molecular markers involved in CRC metastasis in both CTCs and exosomes. Using the GEO, Exocarta, and Vesiclepedia databases, similarities in the expressed genes among the above-mentioned biomarkers were identified. Both gene ontology (GO) analysis and KEGG pathway^15^ were examined to identify key candidate genes, and it was found that *Talin1* could provide significant prognostic value in CRC metastasis.

The evaluation and validation of biological fluids markers as debatable matter should be assessed on tissue samples because of the lack of a consensus over isolation and characterization. Therefore, this step was taken in the current study to optimize the novel findings in the broad range of samples. The mRNA expression of *Talin1* in fresh colorectal tumor samples and their adjacent normal tissues as the control were evaluated using Real-Time PCR. Then, to explore the protein localization and expression level of *Talin1* in CRC samples as well as its correlation with clinicopathological features and survival outcome in CRC patients, the findings from the analysis of gene expression data were validated and confirmed on a large series of CRC tissue microarrays (TMAs) using the immunohistochemistry (IHC) technique.

## Results

### Bioinformatics approach

**Figure 1:**
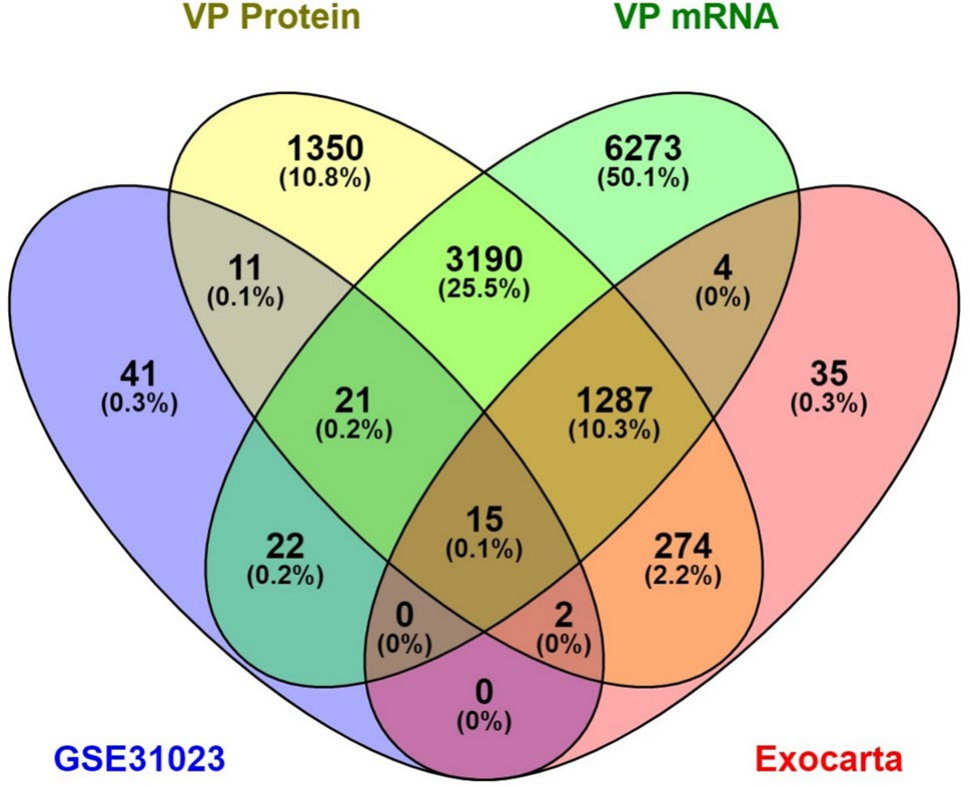
The common molecular markers between CTCs and exosome in databases. Fifteen hub genes were extracted from our mRNA and protein entrance mentioned databases.

To find the valuable molecular marker between CTCs and tumor-derived exosomes that was affected in metastasis after online analysis of the total similarity of their expressed genes, the following 15 genes were found: *TLN1, TMEM51, GNPNAT1, RPS4X, MARCKSL1, FARP1, RHOB, HSP90AB1, LFNG, AHNAK, RPSA, CNPY2, PRDX1, SLC25A5,* and *TGFBI*, as shown in Fig. 1. A detailed analysis of these genes was applied in online web tools such as EnrichR as a collection of databases, and the results based on a *p *value < 0.05 as the cut-off criteria are summarized in Supplementary Table 2.

**Figure 2:**
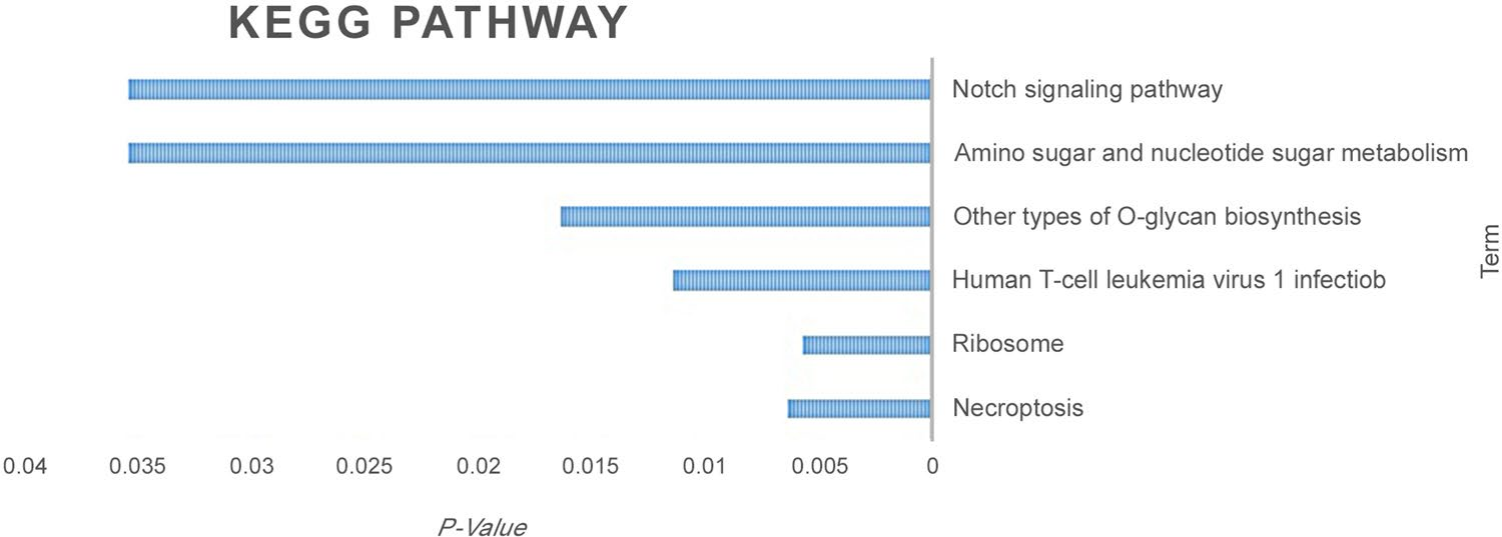
KEGG pathway list related to the 15 excluded genes based on P-Value.

After data analysis of the included genes related to CTCs and exosomes in the literature, it was proven that the overexpression of various integrins can promote cancer cells to be more motile and more invasive. These adhesion molecules can be considered as a major potential breakthrough in oncologic treatment^16,17^, even as a regulators of cancer stemness, metastasis, and drug resistance^18^. *Talin* because of structural and functional significance with integrin cytoplasmic tails resulting in extracellular integrin compartments rearrangements and integrin activation^19^. Specifically, *Talin1,* which functionally contributes to the extravasation and metastasis of colon cancer CTCs^20^, was selected for the current evaluation. *Talin1* protein expression was significantly localized in the cytoplasm that mediates integrin interactions with the extracellular matrix (ECM). From another side, for cell spreading and migration, the link between ECM- bound integrins and intracellular F actin is vital^21^. *Talin1* mediates both inside-out and outside-in signaling, which leads to subsequent cascades^22,23^ and also has a direct effect on the regulation of insulin signaling, actin dynamic, and granule trafficking^24^. *Talin* serves as tension-sensing anchoring as well as a unique mechanotransductive point to link the cells to the ECM. It has been proven using cryoelectron microscopy (cryo-EM) full-length structure examination that the function of *Talin* could be regulated as a two-way mode of auto-inhibition, and the fast switching between its inactive conformations and active could regulate Focal adhesion kinase (FAK) turnover^25^. In our evaluation, focal adhesion among cellular components, cadherin, and RNA binding between molecular function were spotlighted. Also, cytoskeletal anchoring at plasma membrane, platelet aggregation, cell–cell adhesion, and junction throughout the biological process were considered significantly highlighted. Additionally, the KEGG pathway (as shown in Fig. 2) was common, and it was also related to cancer, like *MAPK* and integrin.

**Figure 3:**
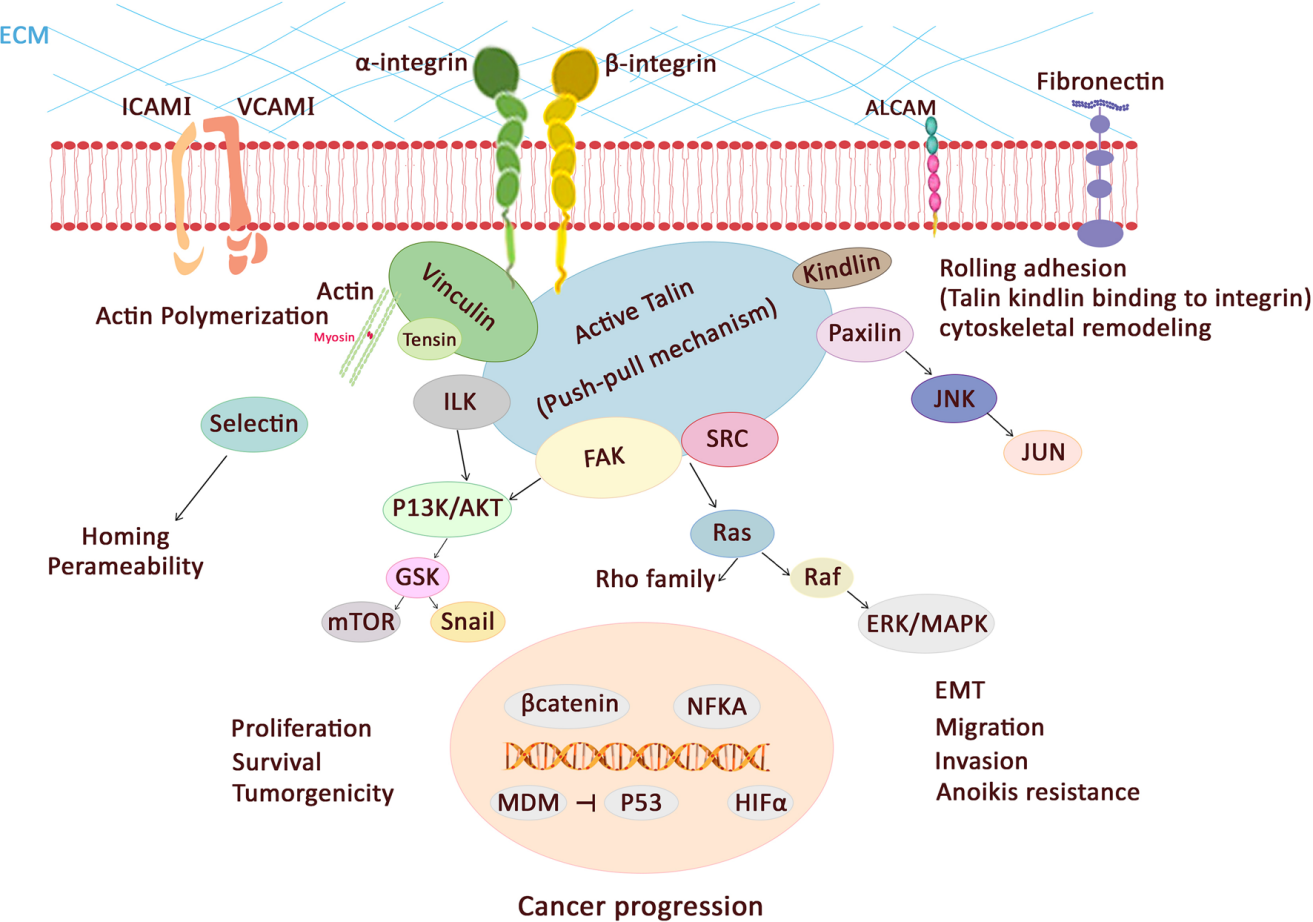
*Talin1* plays significant roles in cancer related pathway.

Overall, the major mediators of such cellular responses are integrins, which link to the actin cytoskeleton via *Talin* as a cytoskeletal protein^26^. *Talin1* plays a key role in malignancies through implication in cell adhesion, migrations, proliferation, invasion, apoptosis, cytoskeleton remodeling, anchorage-independent growth, and tumorigenicity. In Fig. 3, the interaction of *Talin* and other molecules in malignancies are shown.

### Patients’ characteristics of fresh tissue samples

The current study included seventy-seven fresh tissue samples from CRC patients, 52 (67.4%) of which came from males and 25 (32.6%) from females (male/female ratio = 2.12). The median age of patients was 60 years (SD = 14.79, range 20–87); 37 (48%) patients were aged ≤ 60 years and 40 (52%) were aged > 60. Eight (10.4%) cases were diagnosed as stage I, 25 (32.6%) stage II, 27 (35%) and 17 (22%) stage III and IV respectively. Thirty-two (41.6%) cases were low-grade adenocarcinomas, and 14 (18.2%) and 31 (40.2%) were moderated and poorly differentiated adenocarcinomas, respectively.

### Associations between *Talin1* mRNA expression and clinicopathological parameters in fresh tissue samples

**Figure 4:**
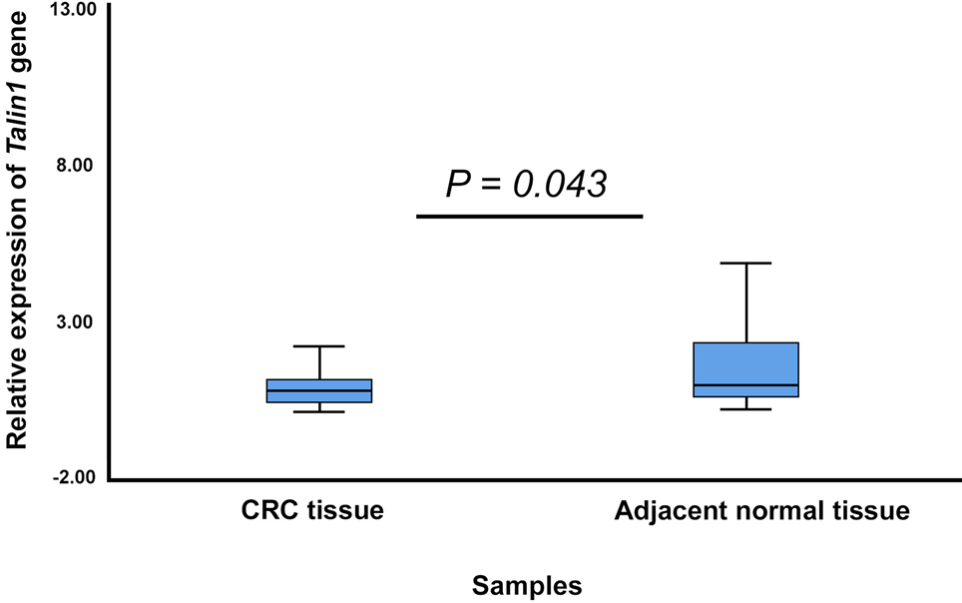
The expression levels of *Talin1* using Real-Time PCR in fresh tissue samples of CRC.

*Talin1* is ubiquitously expressed. Websites like Uniprot^27^ and Genecards^28^ showed it as localized within the cytosol, extracellular, and in the cytoskeleton. The expression profiles of *Talin1* were decreased in patients with CRC compared with the healthy group (*p* < *0.043*) according to Real-Time PCR (Fig. 4).

Pearson’s χ2 test revealed that a statistically significant association among expression levels of *Talin1* mRNA and TNM stage (*p* = *0.034*) as well as gender (*p* = *0.029*), indicating an association between the low level expression of *Talin1* and an increased TNM stage (Table 1).

**Table 1:** The association between mRNA Talin1 expression and clinicopathological parameters of fresh tissue colorectal cancer (CRC) samples.

Patients and tumor characteristics	Total samples N (%)	mRNA Talin1 expression		*P* value
		Low N (%)	High N (%)	
**Median age, years (Range)**				
≤ Median age	37 (48)	24 (31.2)	13 (16.9)	
> Median age	40 (52)	28 (36.3)	12 (15.6)	0.379
**Gender**				
Male	52 (67.4)	39 (50.6)	13 (16.9)	***0.027***
Female	25 (32.6)	24 (31.2)	1 (1.3)	
(Male/Female)	2.12			
**Tumor differentiation**				
Well	32 (41.6)	29 (37.7)	3 (3.9)	0.055
Moderate	14 (18.2)	10 (13)	4 (5.2)	
Poor	31 (40.2)	21 (27.2)	10 (13)	
**TNM stages**				
I	8 (10.4)	7 (9)	1 (1.3)	***0.034***
II	25 (32.6)	23 (29.9)	2 (2.6)	
III	27 (35)	19 (24.7)	8 (10.4)	
IV	17 (22)	15 (19.5)	2 (2.6)	
**Vascular invasion (VI)**				
Present	21 (27.2)	16 (20.7)	5 (6.5)	0.396
Absent	56 (72.8)	47 (61.1)	9 (11.7)	
**Lymph node invasion (LNI)**				
Involved	42 (54.5)	32 (41.6)	10 (13)	0.162
None	35 (45.5)	31 (40.2)	4 (5.2)	
**Neural invasion (NI)**				
Involved	25 (32.6)	22 (28.6)	3 (3.9)	0.347
None	52 (67.4)	41 (53.2)	11 (14.3)	
**Distant metastasis**				
Present	42 (54.5)	32 (41.6)	10 (13)	0.162
Absent	35 (45.5)	31 (40.2)	4 (5.2)	
**Tumor recurrence**				
Yes	7 (9)	7 (9)	0 (0.0)	0.221
No	70 (91)	56 (72.7)	14 (18.3)	

*P* value; Pearson’s χ2 test.

**Figure 5:**
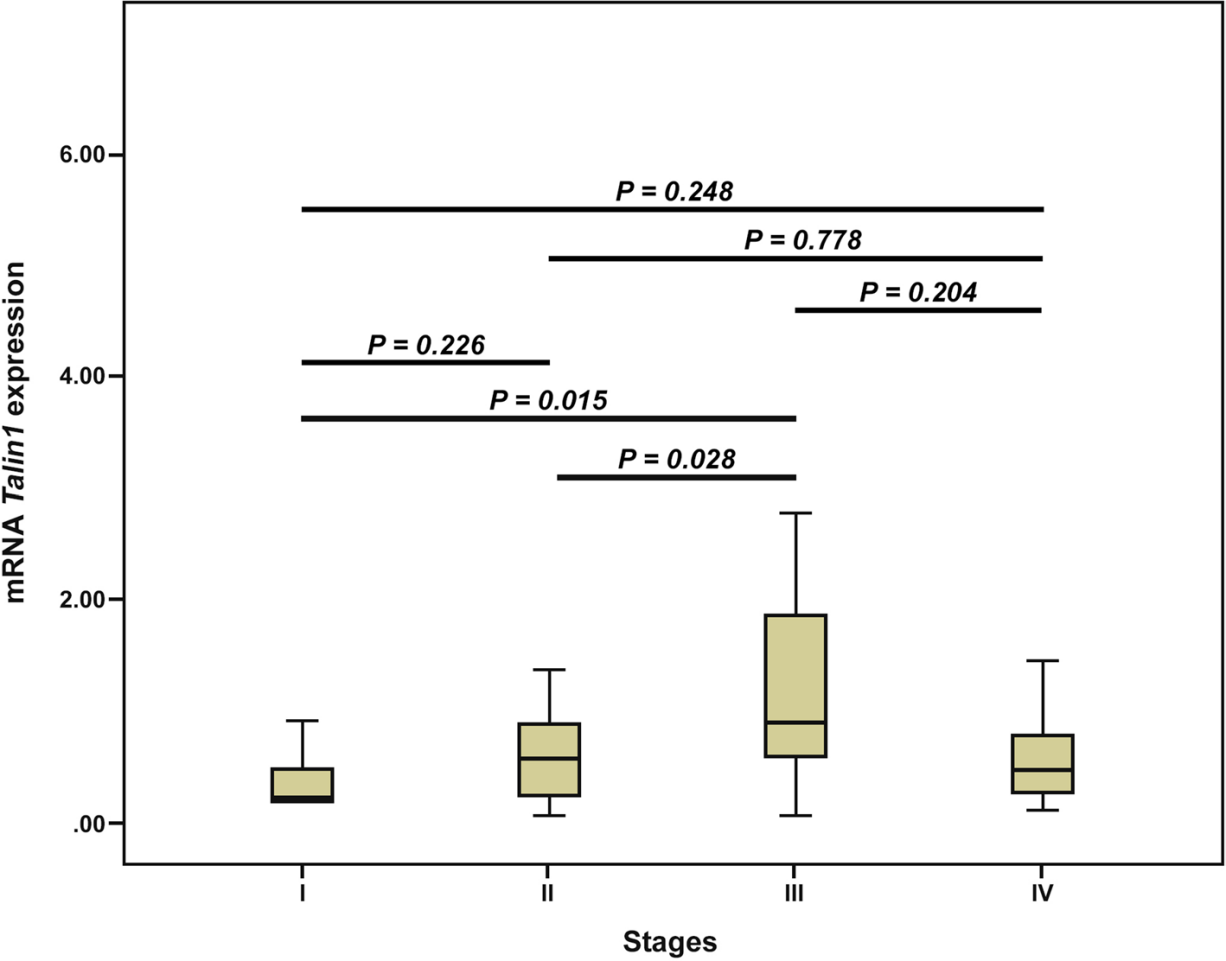
Box plot analysis of mRNA *Talin1* expression levels in stage I to IV. Based on the standard definitions, each box-plot shows the median (bold line) and interquartile lines (box). The result of Mann–Whitney *U* test showed that there is an association for the median of *Talin1* mRNA expression between stages I and III (*p* = *0.015*) as well as stages II and III (*p* = *0.028*). There were no statistically significant differences in the median level of *Talin1* mRNA expression between other stages.

The nonparametric Kruskal–Wallis and Mann–Whitney *U* tests were utilized to measure the differences between the median expressions of *Talin1* among the groups. The results of the Mann–Whitney *U* test also showed a statistically significant difference in the median level of *Talin1* mRNA expression between stages I and III (*p* = *0.015*) and stages II and III (*p* = *0.028*). Further, we did not observe any statistically significant differences in the median level of *Talin1* mRNA expression between other stages (Fig. 5). No association was found between *Talin1* mRNA expressions or with other clinicopathological characteristics (Table 1).

### Prognostic value of *Talin1* mRNA expression for clinical outcome in CRC patients

From the 77 CRC samples, 70 (91%) patients had no history of recurrence, while 7 (9%) patients were positive. In 42 (54.5%) metastasis occurred, while 35 (45.5%) patients were negative for it. Thirty (39.0%) patients had no history of metastasis, recurrence, or cancer-related death, while 47 (61.0%) patients for these parameters were positive. During the follow-up period, cancer-related death in 26 (33.8%) patients was reported. The mean duration of follow-up time was 20 months (SD = 11.3(, median was 25 months (8, 28), and the range was from 1 to 33 months. The mean DSS time for patients with high and low expression of *Talin1 mRNA* were 23.8 (SD = 1.6) and 27.2 (SD = 3.4) months, respectively.

**Figure 6:**
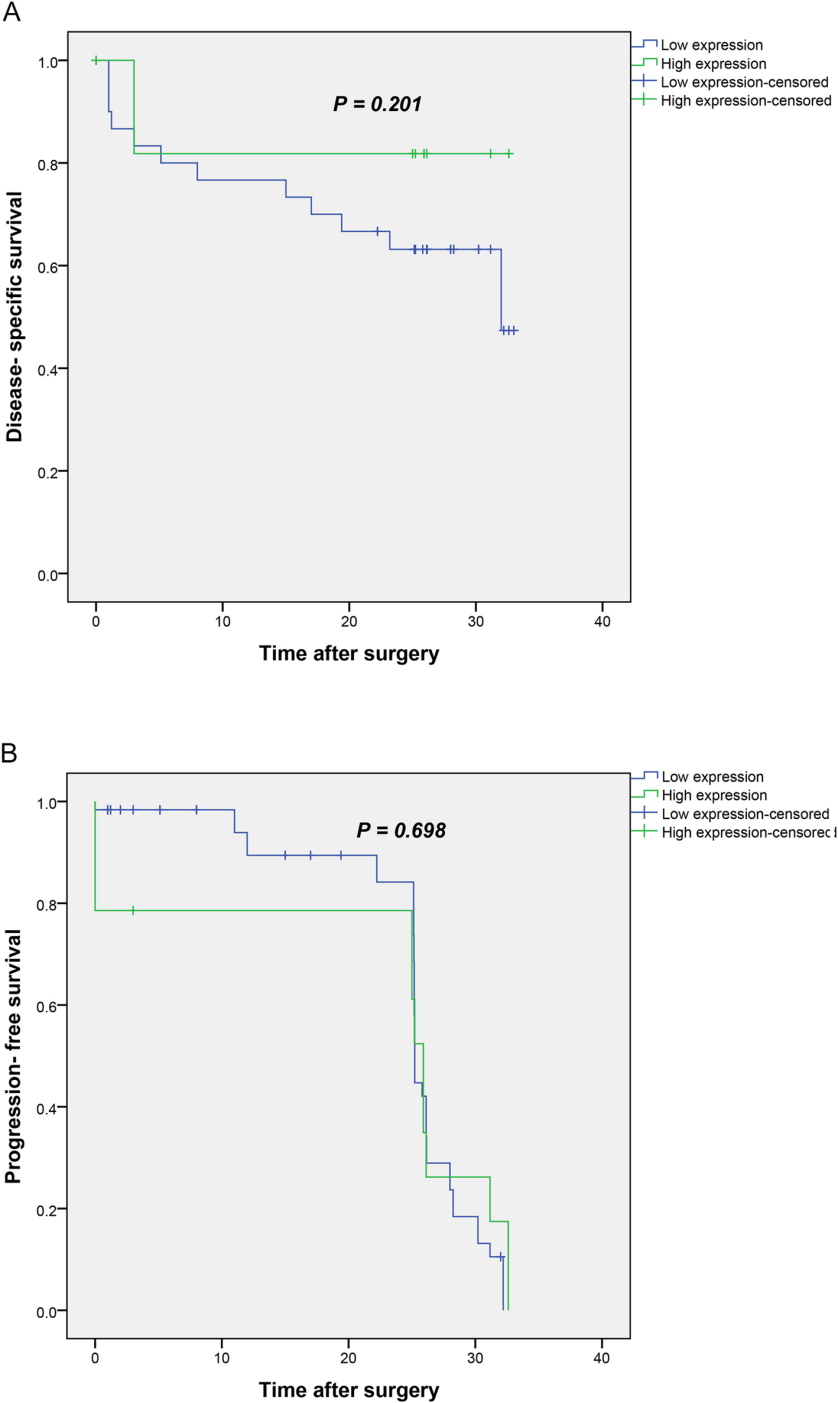
(**A**) Kaplan–Meier curves for disease-specific survival (DSS) and progression-free survival (PFS) based on mRNA *Talin1* expression levels in CRC patients. In CRC patients, lower level of *Talin1* mRNA expression was associated with shorter DSS compared to the tumors with high expression of *Talin1* mRNA expression (*P* = 0.201). (**B**) Kaplan–Meier curve showed that low level of *Talin1* expression is not significantly related to PFS (*P* = 0.698).

The results of the Kaplan–Meier curve demonstrated that patients with low *Talin1* mRNA expression had shorter DSS compared to patients with high expression of *Talin1* but without a statistically significant association (Log Rank test, *p* = 0.201) (Fig. 6A).

Furthermore, the mean PFS time for patients with low and high expression rates of *Talin1 mRNA* were 24.9 (SD = 0.89) and 21.7 (SD = 3.2) months, respectively. The Kaplan–Meier curve showed that low level *Talin1* expression is not significantly related to PFS (*p* = 0.698) (Fig. 6B).

### Characteristics of patients’ FFPE tissue samples

Because the application of tissue microarray (TMA) technology has covered the evaluation of clinical values of huge number of candidate markers, 760 cases were included in this research, and for final scoring, 648 CRCs tissues were left. The FFPE population samples consisted of 303 (46.8%) male and 262 (40.4%) female patients, with a male/female ratio of 1.15. The median age of patients was calculated as 60 years (SD = 14.79, range 23–92). To clarify, 298 (46.0%) were ≤ 60 and 267 (41.2%) were > 60. Tumor size ranged from 1 to 20 cm, and tumors were classified into two groups. Three hundred and eighty-two (59.0%) of cases had tumors that were smaller in size than the mean (size ≤ 4.5) and 181 (27.9%) had tumors larger than the mean (size > 4.5). In this study, 90 (13.9%) patients had stage I, 254 (39.2%) stage II, 198 (30.6%) stage III, and 22 (3.4%) stage IV tumors. Moreover, 212 (32.7%) cases were grade I, 316 (48.8%) were grade II, and 34 (5.2%) were grade III; VI was found in 87 (13.4%) cases, and LNI and NI were observed in 212 (32.7%) cases. Tumor recurrence was seen in 96 (14.8%) patients, and 90 (13.9%) patients showed distant metastasis during the follow-up period.

### Expression of *Talin1* protein in CRC and adjacent normal tissues samples

**Figure 7:**
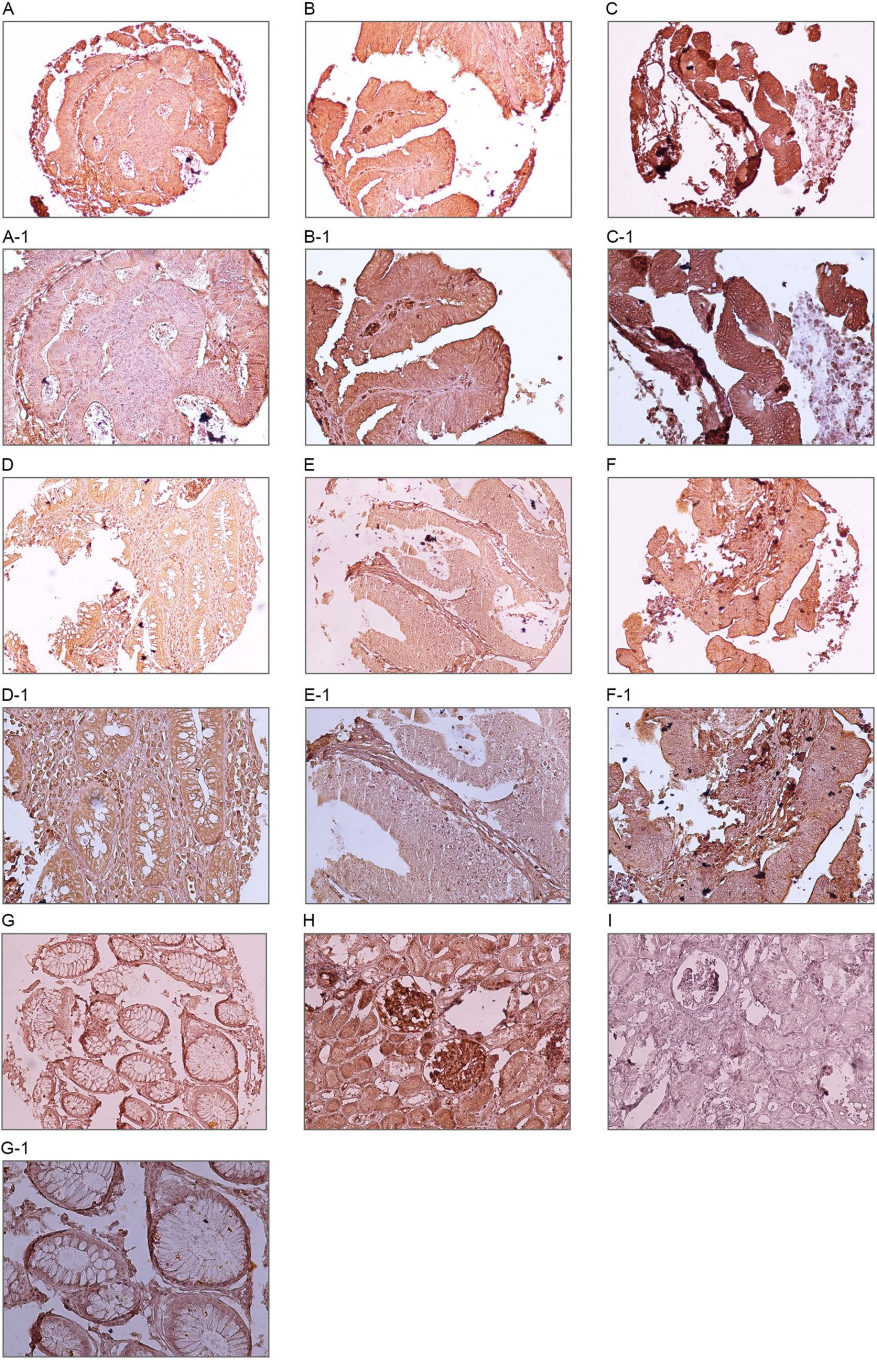
Immunohistochemical (IHC) analysis of *Talin1* expression in different colorectal cancer (CRC) samples. CRC samples expressed *Talin1* at various levels. Cytoplasmic expression of *Talin1* at various levels: weak (**A**), moderate (**B**), and strong (**C**) in magnification of × 200; Weak (**A-1**), moderate (**B-1**), and strong (**C-1**) in magnification × 400. ECM expression of *Talin1* in CRC at various levels is as follows: weak (**D**), moderate (**E**), and strong (**F**) in magnification × 200. Weak (**D-1**), moderate (**E-1**), and strong (**F-1**) in magnification × 400. IHC staining of kidney tissue was presented as positive (**H**) and negative (**I**) controls, adjacent normal tissue sample **(G, G-1)** in magnification × 200; and × 400.

The expression level of *Talin1* protein was evaluated using IHC on TMA sections by three scoring methods. H-score consist of staining intensity and percentage of positive tumor cells. In the CRC samples, *Talin1* was expressed at varying intensities in the cell cytoplasm and ECM. Cytoplasmic and ECM expressions of *Talin1* were detected in 643 (99.2%) and 628 (96.9%) CRC cases, respectively. The mean expression of *Talin1* was 149 in cytoplasm and 138 in ECM. *Talin1* expression based on the H-score demonstrated that low, moderate, and high levels of cytoplasmic expression of *Talin1* were observed in 205 (31.6%), 349 (53.9%), and 94 (14.5%) CRC samples, respectively. Moreover, low, moderate, and high ECM expression of *Talin1* was found in 235 (36.3%), 379 (58.5%), and 34 (5.2%) CRC patients, respectively (Table 2). The expression of *Talin 1* was observed in the cytoplasm and ECM in all cases of adjacent normal tissue (Table 2). The mean expression of *Talin1* was 151 in cytoplasm and 161 in ECM; therefore, *Talin1* expression was greater in the adjacent normal tissue samples than in CRC samples, particularly in ECM expression. Moreover, human kidney tissue, showed strong staining as a positive control (Fig. 7).

**Table 2:** Cytoplasmic and extra cellular matrix (ECM) Talin1 expression (Intensity of staining, percentage of positive tumor cells, and H-score in FFPE colorectal cancer (CRC) and adjacent normal tissues.

Scoring system	Cytoplasmic expression N (%)		ECM expression N (%)	
	CRC samples	Adjacent normal tissues	CRC samples	Adjacent normal tissues
**Intensity of staining**				
Negative (0)	5 (0.8)	24 (35.8)	20 (3.1)	10 (14.9)
Weak (+ 1)	187 (28.9)	33 (49.3)	189 (29.2)	50 (74.6)
Moderate (+ 2)	361 (55.7)	3 (4.5)	404 (62.3)	0 (0.0)
Strong (+ 3)	95 (14.7)	0 (0.0)	35 (5.4)	0 (0.0)
Not identified	0 (0.0)	7 (10.4)	0 (0.0)	7 (10.4)
**Percentage of positive tumor cells**				
< 25%	18 (2.8)	0 (0.0)	30 (4.6)	0 (0.0)
25–50%	25 (3.9)	0 (0.0)	51 (7.9)	1 (1.5)
51- 75%	156 (24.1)	6 (9.0)	162 (25.0)	11 (16.4)
> 75%	449 (69.3)	54 (80.6)	405 (62.5)	48 (71.6)
Not identified	0 (0.0)	7 (10.4)	0 (0.0)	7 (10.4)
**H-score (3 groups)**				
0–100	205 (31.6)	25 (37.3)	235 (36.3)	11 (16.4)
101–200	349 (53.9)	32 (47.8)	379 (58.5)	49 (73.1)
201–300	94 (14.5)	3 (4.5)	34 (5.2)	0 (0.0)
Not identified	0 (0.0)	7 (10.4)	0 (0.0)	7 (10.4)
Total	648 (100)	67 (100)	648 (100)	67 (100)

H-score indicates Histological score.

### Associations between Expression of *Talin1* protein and clinicopathological characteristics in CRC patients

**Figure 8:**
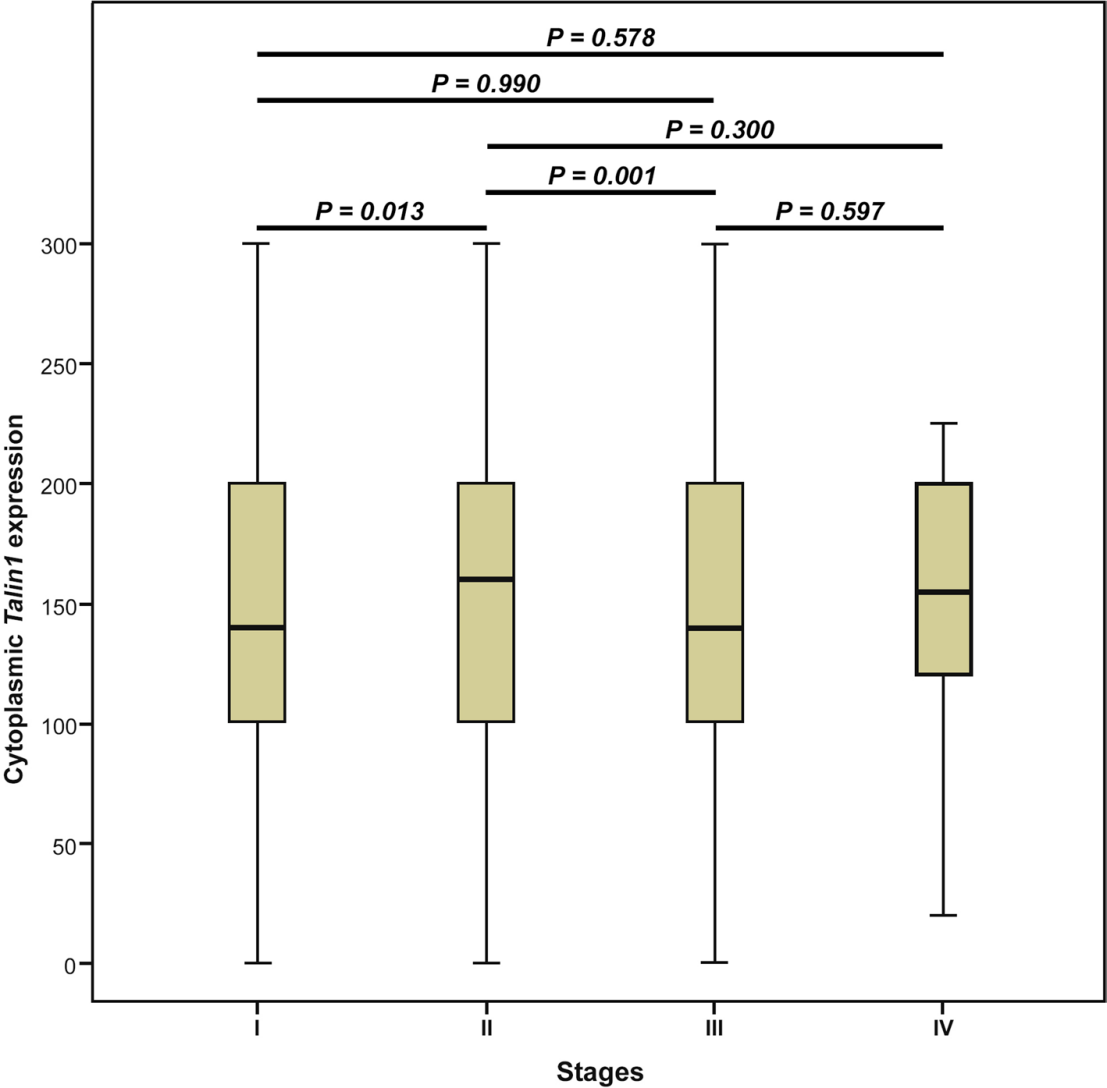
Box plot analysis of *Talin1* expression levels in stage I to IV. Based on the standard definitions, each box-plot shows the median (bold line) and interquartile lines (box). The result of Mann–Whitney *U* test showed that there is a statistically significant association for the median of cytoplasmic *Talin1* expression between stages I and II (*P* = *0.013*) as well as stages II and III (*P* = *0.001*). There were no statistically significant differences in the median level of *Talin1* protein expression between other stages.

The results of Pearson’s χ2 test showed that there is an association between expression levels of *Talin1* and TNM stage (*p* = *0.028*) as well as gender (*p* = *0.009*) (Table 3). The non-parametric Kruskal–Wallis test indicated a difference between the median expression level of *Talin1* protein and various TNM stages (I–IV) (*p* = *0.004*). The results of the Mann–Whitney *U* test revealed a statistically significant difference in the median level of *Talin1* protein expression between stages I and II (*p* = *0.013*) and stages II and III (*p* = *0.001*). We did not find any statistically significant differences in the median level of *Talin1* protein expression between other stages of CRC cases (Fig. 8).

**Table 3:** The association between cytoplasmic Talin1 expression and clinicopathological parameters of colorectal cancer (CRC) samples (Intensity of staining and H-score).

Patients and tumor characteristics	Total samples N (%)	Intensity of staining N (%)				*P* value	H-score N (%)			*P* value
		0 (Negative)	1 + (Weak)	2 + (Moderate)	3 + (Strong)		H-score 0–100	H-score 101–200	H-score 201–300	
Median age, years (Range)	60 (23–92)									
≤ Median age	298 (46.0)	2 (50.0)	87 (50.9)	161 (51.8)	48 (60.8)	0.492	95 (51.6)	156 (51.5)	47(60.3)	0.359
> Median age	267 (41.2)	2 (50.0)	84 (49.1)	150 (48.2)	31 (39.2)		89 (48.4)	147 (48.5)	31 (39.7)	
Not identified	83 (12.8)	0 (0.0)	0 (0.0)	0 (0.0)	0 (0.0)		0 (0.0)	0 (0.0)	0 (0.0)	
**Gender**										
Male	303 (46.8)	0 (0.0)	93 (54.4)	157 (50.5)	53 (67.1)	***0.009***	96 (52.2)	154 (50.8)	53 (67.9)	***0.023***
Female	262 (40.4)	4 (100.0)	78 (45.6)	154 (49.5)	26 (32.9)		88 (47.8)	149 (49.2)	25 (32.1)	
Not identified (Male/Female)	83 (12.8) 1.15	0 (0.0)	0 (0.0)	0 (0.0)	0 (0.0)		0 (0.0)	0 (0.0)	0 (0.0)	
Mean tumor size (cm)	4.5 (1–20)									
≤ Mean	382 (59.0)	2 (50.0)	115 (67.6)	212 (68.2)	53 (67.9)	0.896	123 (67.2)	207 (68.3)	52 (67.5)	0.967
> Mean	181 (27.9)	2 (50.0)	55 (32.4)	99 (31.8)	25 (32.1)		60 (32.8)	96 (31.7)	25 (32.5)	
Not identified	85 (13.1)	0 (0.0)	0 (0.0)	0 (0.0)	0 (0.0)		0 (0.0)	0 (0.0)	0 (0.0)	
**Tumor differentiation**										
Well	212 (32.7)	1 (25.0)	66 (38.8)	113 (36.5)	32 (41.0)	0.832	68 (37.2)	112 (37.1)	32 (41.6)	
Moderate	316 (48.8)	3 (75.0)	91 (53.5)	179 (57.7)	43 (55.1)		102 (55.7)	172 (57.0)	42 (54.5)	0.852
Poor	34 (5.2)	0 (0.0)	13 (7.6)	18 (5.8)	3 (3.8)		13 (7.1)	18 (6.0)	3 (3.9)	
Not identified	86 (13.3)	0 (0.0)	0 (0.0)	0 (0.0)	0 (0.0)		0 (0.0)	0 (0.0)	0 (0.0)	
**TNM stages**										
I	90 (13.9)	1 (25.0)	32 (18.7)	46 (14.8)	11 (14.1)	0.254	35 (19.0)	44 (14.5)	11 (14.3)	
II	254 (39.2)	1 (25.0)	66 (38.6)	143 (46.0)	44 (56.4)		68 (37.0)	142 (46.9)	44 (57.1)	***0.028***
III	198 (30.6)	2 (50.0)	68 (39.8)	106 (34.1)	22 (28.2)		76 (41.3)	101 (33.3)	21 (27.3)	
IV	22 (3.4)	0 (0.0)	5 (2.9)	22 (28.2)	1 (1.3)		5 (2.7)	16 (5.3)	1 (1.3)	
Not identified	84 (13.0)	0 (0.0)	0 (0.0)	0 (0.0)	0 (0.0)		0 (0.0)	0 (0.0)	0 (0.0)	
**Vascular invasion (VI)**										
Present	87 (13.4)	1 (25.0)	24 (14.0)	49 (15.8)	13 (16.7)	0.884	28 (15.2)	47 (15.5)	12 (15.6)	0.995
Absent	477 (73.6)	3 (75.0)	147 (86.0)	262 (84.2)	65 (83.3)		156 (84.8)	256 (84.5)	65 (84.4)	
Not identified	84 (13.0)	0 (0.0)	0 (0.0)	0 (0.0)	0 (0.0)		0 (0.0)	0 (0.0)	0 (0.0)	
**Lymph node invasion (LNI)**										
Involved	212 (32.7)	2 (50.0)	71 (41.5)	114 (36.7)	25 (32.1)	0.471	79 (42.9)	109 (36.0)	24 (31.2)	0.14
None	352 (54.3)	2 (50.0)	100 (58.5)	197 (63.3)	53 (67.9)		105 (57.1)	194 (64.0)	53 (68.8)	
Not identified	84 (13.0)	0 (0.0)	0 (0.0)	0 (0.0)	0 (0.0)		0 (0.0)	0 (0.0)	0 (0.0)	
**Neural invasion (NI)**										
Involved	212 (32.7)	1 (25.0)	33 (19.3)	71 (22.8)	10 (12.8)	0.254	36 (19.6)	70 (23.1)	9 (11.7)	0.08
None	352 (54.3)	3 (75.0)	138 (80.7)	240 (77.2)	68 (87.2)		148 (80.4)	233 (76.9)	68 (88.3)	
Not identified	84 (13.0)	0 (0.0)	0 (0.0)	0 (0.0)	0 (0.0)		0 (0.0)	0 (0.0)	0 (0.0)	
**Distant metastasis**										
Present	90 (13.9)	2 (40.0)	30 (28.0)	48 (26.2)	10 (18.5)	0.509	35 (29.2)	46 (26.1)	9 (17.0)	0.238
Absent	259 (40.0)	3 (60.0)	77 (72.0)	135 (73.8)	44 (81.5)		85 (70.8)	130 (73.9)	44 (83.0)	
Not identified	299 (46.1)	0 (0.0)	0 (0.0)	0 (0.0)	0 (0.0)		0 (0.0)	0 (0.0)	0 (0.0)	
**Tumor recurrence**										
Yes	96 (14.8)	2 (40.0)	28 (26.2)	55 (30.1)	11 (20.0)	0.45	33 (27.5)	53 (30.1)	10 (18.5)	0.248
No	254 (39.2)	3 (60.0)	79 (73.8)	128 (69.9)	44 (80.0)		87 (72.5)	123 (69.9)	44 (81.5)	
Not identified	298 (46.0)	0 (0.0)	0 (0.0)	0 (0.0)	0 (0.0)		0 (0.0)	0 (0.0)	0 (0.0)	

H-score indicates Histological score.

Values in bold are statistically significant.

*P* value; Pearson’s χ2 test.

Further, the results of Spearman’s correlation exhibited a significant adverse correlation between *Talin1* protein expression and advanced TNM stages (*p* = 0.038). No association was found between cytoplasmic *Talin1* protein expression and the other clinicopathological characteristics (Table 3).

The results of Pearson’s χ2 and Spearman’s correlation tests showed that there is no association or correlation between ECM *Talin1* expression and the clinicopathological parameters of CRCs patients (Table 4).

**Table 4:** The association between extra cellular matrix (ECM) Talin1 expression and clinicopathological parameters of colorectal cancer (CRC) samples (Intensity of staining and H-score).

Patients and tumor characteristics	Total samples N (%)	Intensity of staining N (%)				*P* value	H-score N (%)			*P* value
		0 (Negative)	1 + (Weak)	2 + (Moderate)	3 + (Strong)		H-score 0–100	H-score 101–200	H-score 201–300	
Median age, years (Range)	60 (23–92)									
≤ Median age	298 (46.0)	14 (73.7)	82 (49.1)	187 (54.0)	15 (45.5)	0.160	109 (52.9)	174 (53.2)	15 (46.9)	0.789
> Median age	267 (41.2)	5 (26.3)	85 (50.9)	159 (46.0)	18 (54.5)		97 (47.1)	153 (46.8)	17 (53.1)	
Not identified	83 (12.8)	0 (0.0)	0 (0.0)	0 (0.0)	0 (0.0)		0 (0.0)	0 (0.0)	0 (0.0)	
**Gender**										
Male	303 (46.8)	6 (31.6)	82 (49.1)	195 (56.4)	20 (60.6)	0.79	99 (48.1)	185 (56.6)	19 (59.4)	0.126
Female	262 (40.4)	23 (68.4)	85 (50.9)	151 (43.6)	13 (39.4)		107 (51.9)	142 (43.4)	13 (40.6)	
Not identified (Male/Female)	83 (12.8) 1.15	0 (0.0)	0 (0.0)	0 (0.0)	0 (0.0)		0 (0.0)	0 (0.0)	0 (0.0)	
Mean tumor size (cm)	4.5 (1–20)									
≤ Mean	382 (59.0)	11 (57.9)	118 (70.7)	231 (67.2)	22 (66.7)	0.667	141 (68.4)	220 (67.7)	21 (65.6)	0.947
> Mean	181 (27.9)	8 (42.1)	49 (29.3)	113 (32.8)	11 (33.3)		65 (31.6)	105 (32.3)	11 (34.4)	
Not identified	85 (13.1)	0 (0.0)	0 (0.0)	0 (0.0)	0 (0.0)		0 (0.0)	0 (0.0)	0 (0.0)	
**Tumor differentiation**										
Well	212 (32.7)	6 (31.6)	64 (38.3)	128 (37.3)	14 (42.4)	0.952	81 (39.3)	117 (36.1)	14 (43.8)	
Moderate	316 (48.8)	12 (63.2)	91 (54.5)	195 (56.9)	18 (54.5)		112 (54.4)	187 (57.7)	17 (53.1)	0.833
Poor	34 (5.2)	1 (5.3)	12 (7.2)	20 (5.8)	1 (3.0)		13 (6.3)	20 (6.2)	1 (3.1)	
Not identified	86 (13.3)	0 (0.0)	0 (0.0)	0 (0.0)	0 (0.0)		0 (0.0)	0 (0.0)	0 (0.0)	
**TNM stages**										
I	90 (13.9)	0 (0.0)	35 (21.0)	49 (14.2)	6 (18.2)	0.199	38 (18.4)	46 (14.1)	6 (18.8)	
II	254 (39.2)	10 (52.6)	69 (41.3)	159 (46.1)	16 (48.5)		88 (42.7)	151 (46.3)	15 (46.9)	0.252
III	198 (30.6)	7 (36.8)	54 (32.3)	127 (36.8)	10 (30.3)		67 (32.5)	121 (37.1)	10 (31.3)	
IV	22 (3.4)	2 (10.5)	9 (5.4)	10 (2.9)	1 (3.0)		13 (6.3)	8 (2.5)	1 (3.1)	
Not identified	84 (13.0)	0 (0.0)	0 (0.0)	0 (0.0)	0 (0.0)		0 (0.0)	0 (0.0)	0 (0.0)	
**Vascular invasion (VI)**										
Present	87 (13.4)	3 (15.8)	25 (15.0)	56 (16.2)	3 (9.1)	0.749	32 (15.5)	52 (16.0)	3 (9.4)	0.616
Absent	477 (73.6)	16 (84.2)	142 (85.0)	289 (83.8)	30 (90.9)		174 (84.5)	274 (84.0)	29 (90.6)	
Not identified	84 (13.0)	0 (0.0)	0 (0.0)	0 (0.0)	0 (0.0)		0 (0.0)	0 (0.0)	0 (0.0)	
**Lymph node invasion (LNI)**										
Involved	212 (32.7)	11 (57.9)	60 (35.9)	130 (37.7)	11 (33.3)	0.285	79 (38.3)	122 (37.4)	11 (34.4)	0.907
None	352 (54.3)	8 (42.1)	107 (64.1)	215 (62.3)	22 (66.7)		127 (61.7)	204 (62.6)	21 (65.6)	
Not identified	84 (13.0)	0 (0.0)	0 (0.0)	0 (0.0)	0 (0.0)		0 (0.0)	0 (0.0)	0 (0.0)	
**Neural invasion (NI)**										
Involved	212 (32.7)	5 (26.3)	32 (19.2)	76 (22.0)	2 (6.1)	0.15	42 (20.4)	71 (21.8)	2 (6.3)	0.115
None	352 (54.3)	14 (73.7)	135 (80.8)	269 (78.0)	31 (93.9)		164 (79.6)	255 (78.2)	30 (93.8)	
Not identified	84 (13.0)	0 (0.0)	0 (0.0)	0 (0.0)	0 (0.0)		0 (0.0)	0 (0.0)	0 (0.0)	
**Distant metastasis**										
Present	90 (13.9)	5 (35.7)	29 (26.9)	54 (26.0)	2 (10.5)	0.376	37 (26.8)	51 (26.6)	2 (10.5)	0.294
Absent	259 (40.0)	9 (64.3)	79 (73.1)	154 (74.0)	17 (89.5)		101 (73.2)	141 (73.4)	17 (89.5)	
Not identified	299 (46.1)	0 (0.0)	0 (0.0)	0 (0.0)	0 (0.0)		0 (0.0)	0 (0.0)	0 (0.0)	
**Tumor recurrence**										
Yes	96 (14.8)	5 (35.7)	31 (28.7)	53 (25.5)	7 (35.0)	0.672	39 (28.3)	50 (26.0)	7 (35.0)	0.667
No	254 (39.2)	9 (64.3)	77 (71.3)	155 (74.5)	13 (65.0)		99 (71.7)	142 (74.0)	13 (65.0)	
Not identified	298 (46.0)	0 (0.0)	0 (0.0)	0 (0.0)	0 (0.0)		0 (0.0)	0 (0.0)	0 (0.0)	

H-score indicates Histological score.

Values in bold are statistically significant.

*P* value; Pearson’s χ2 test.

### Prognostic value of *Talin1* protein expression for clinical outcome in CRC

Out of 648 CRC samples included in the present study, survival analysis was performed on 350 patients for whom follow-up data was accessible. From these 350 patients, 239 (68.3%) patients had no history of metastasis, recurrence, or cancer-related death, while 111 (31.7%) patients for these parameters were positive. In 91 (26.0%) patients metastasis and in 96 (27.4%) ones recurrence occurred. In 97 (27.7%), patients cancer-related death was documented during the mean duration of 45.0 months (SD = 27.6) as follow-up time with a median of 41.0 months (27, 66); the range was from 1 to 117 months.

### Survival outcomes based on expression of *Talin1* in CRC

**Figure 9:**
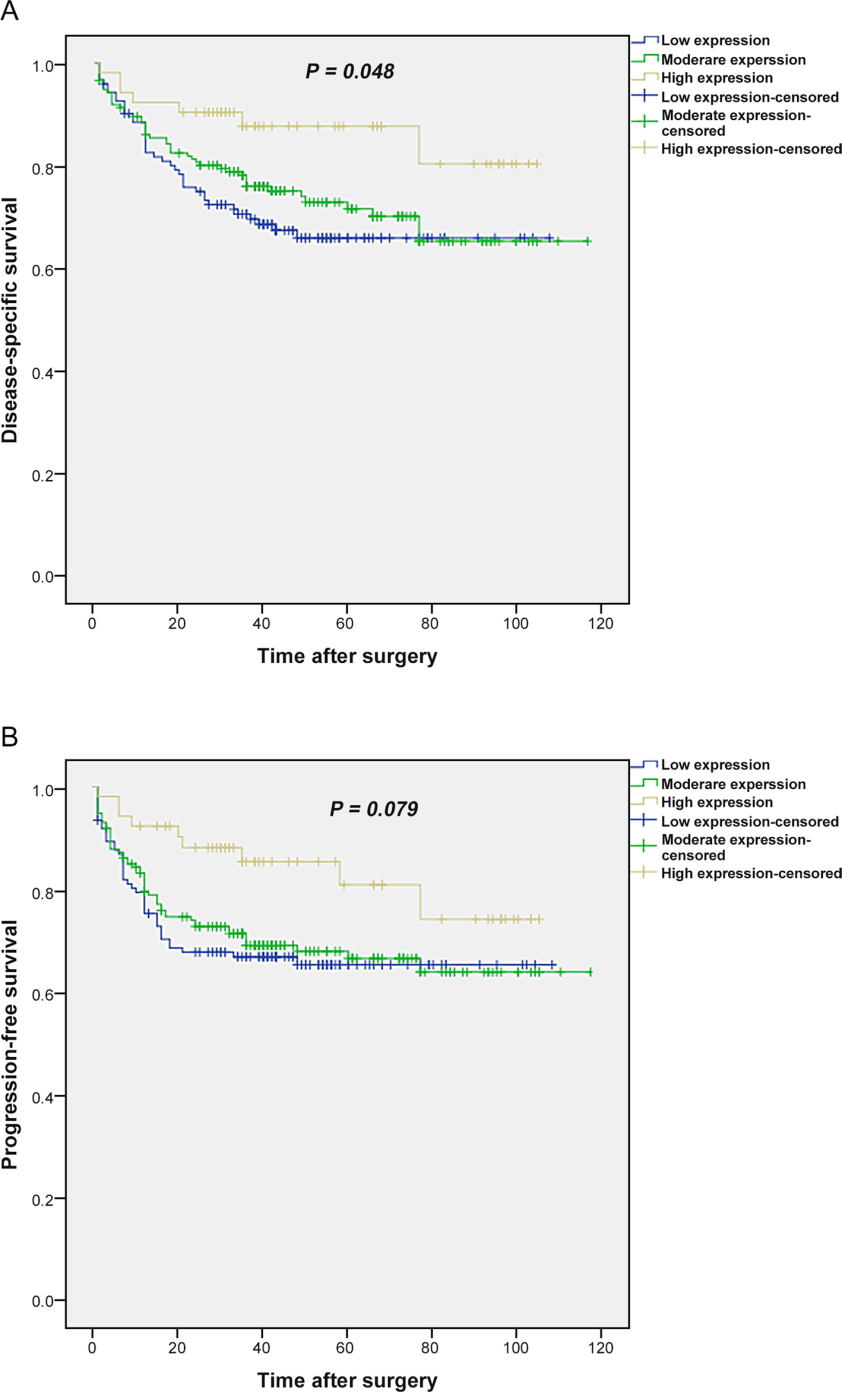
Kaplan–Meier curves for disease-specific survival (DSS) and progression-free survival (PFS) based on cytoplasmic *Talin1* protein expression level in CRC patients. (**A**) In CRC patients, lower level of *Talin1* protein expression was associated with shorter DSS compared to the tumors with high expression of this protein (*P* = 0.048). (**B**) Kaplan–Meier survival analysis showed that low levels of *Talin1* protein expression are not significantly related to PFS (*P* = 0.079).

Kaplan–Meier curve results indicated significant differences between the patients with three groups of cytoplasmic expression rates of *Talin1* and DSS (Log Rank test, *p* = *0.048*) (Fig. 9A). The mean DSS times for patients with high, moderate, and low cytoplasmic expression of *Talin1* were 91 (SD = 4.6), 86 (SD = 3.7), and 47 (SD = 4.0) months, respectively. Furthermore, the 5-year survival rates for DSS in patients whose samples expressed three groups of cytoplasmic *Talin1* expression was 88%, 72%, and 65%, respectively (*p* = 0.039). Besides, there was no significant differences between PFS and patients with cytoplasmic *Talin1* expression levels (*p* = 0.079) (Fig. 9B). The mean PFS times for patients with three groups of cytoplasmic expression of *Talin1* were 87 (SD = 5.1), 81 (SD = 3.9), and 74 (SD = 4.3) months, respectively.

Univariate and multivariate analyses were performed to investigate whether *Talin1* expression was an independent prognostic factor of DSS or PFS and to assess the clinical significance of various parameters that might influence survival outcomes in CRC patients^29^. Among all of the clinicopathological parameters, only cytoplasmic *Talin1* expression (*p* = 0.050), LNI (*p* = 0.010), NI (*p* = 0.008), distant metastasis (*p* < 0.001), tumor recurrence (*p* < 0.001), and age (*p* = 0.009) affecting DSS in univariate analysis. In addition, in multivariate analysis, only age (*p* = 0.015) was significantly related to DSS in these cases (Table 5).

**Table 5:** Univariate and multivariate Cox regression analyses of potential prognostic factor for disease-specific survival (DSS) in patients with colorectal cancer.

Covariate	Univariate analysis		Multivariate analysis	
	HR (95% CI)	P value	HR (95% CI)	P value
H-score		0.050		0.174
High versus Low	0.814 (0.532–1.245)	0.342	0.909 (0.582–1.418)	0.673
High versus Moderate	0.378 (0.169–0.847)	0.018	0.437 (0.183–1.042)	0.062
Age	1.780 (1.155–2.743)	0.009	1.717 (1.113–2.649)	0.015
Lymph node invasion (LNI)	0.571 (0.372–0.877)	0.010	–	–
Neural invasion (NI)	0.523 (0.324–0.845)	0.008	–	–
Distant metastasis	0.104 (0.066–0.162)	< 0.001	–	–
Tumor recurrence	0.131 (0.084–0.203)	< 0.001	–	–

H-score indicates Histological score.

Values in bold are statistically significant.

The variables with *P* value less than 0.05 were included in multivariable analyses.

*HR* hazard ratio, *CI* confidence interval.

Kaplan–Meier analysis showed that there were no differences between DSS or PFS and the patients with three groups of ECM expression of *Talin1* (Log Rank test; *p* = 0.815, *p* = 0.595), respectively. Moreover, the results of univariate and multivariate analyses indicated that the listed clinicopathologic variables were not affecting the DSS or PFS of CRC patients.

## Discussion

Because cross-talking between cancer cells and exosomes plays a vital role in the CRC dynamic network, especially in metastasis^30^, the identification and characterization of both molecular markers as powerful tools in non-invasive cancer monitoring are significant steps to improving our understanding of molecular and cellular mechanisms of cancer metastasis^31^. Furthermore, the identification of new prognostic markers for the likelihood of metastasis that can stratify patients into various classification is clearly warranted. Therefore, considering the heterogeneity and complexity of CRC, this study was conducted to predict prognoses of CRC patient based on the evaluation of their gene expression network and to the clinical significance of their common molecular marker by bioinformatics analysis in multiple fashions. We take advantage of the limited available data related to CTC and exosome collection assays throughout online web tools for tracking CRC metastasis. “Wet-lab” experiments using patient specimens for identifying cancer biomarkers through bioinformatics analysis were considered vital. The best application of that can be develop a practically manageable gene based on applying *p* values and enrichment pathway analysis^32^.

Traditional biomarkers (CEA, CA19-9 and FOBT), as well as colon/sigmoidoscopy, play an unsatisfactory specificity role in CRC screening^7^. Since the limitation of these various methods are considerable^33^ shifting to CTCs and TDEs as a liquid biopsy approach^34,35^ attracted much attention and help to discover markers which are important in diagnostic, prognostic and cancer staging^10,36,37^, estimation of relapse risk, therapeutic targets identification^38,39^, and proper monitoring of patients^8,40^. Regardless of the metastatic site, CTC enumeration are enough as a proper cancer-monitoring index whenever *CEA* and other marker levels are not measurable^41,42^.

Although several mechanisms are involved in cancer progression, recent evidence points to EMT and CSCs as having significant roles in these processes^43^. EMT plays an important role in the invasion and metastasis of CRC, which is mediated by FAK. However, whether FAK participates in EMT, through the EGF/EGFR signaling pathway, remains unknown^44^. *Talin1* can activate integrin's as core regulators^45^ and play an important role as a focal adhesion proteins as well as be mediators of adhesion to the ECM^46^. Therefore, it must be considered extensively to determine its role clearly. FAK-*Talin* binding is required for adhesion turnover and cell motility and in our view has a significant point in common with molecules between circulating tumor cells (CTCs) and tumor-derived exosomes (TDEs).

In malignant tumors, EMT is crucial for acquisition of a mesenchymal phenotype with invasive and metastatic properties leading to tumor progression. The important markers related to this process include *Twist *^47–49^*, E-cadherin, vimentin, N-cadherin, Snail, Slug, integrin's, cytokeratin's, fibronectin, b-catenin, ZEB1, ZEB2, and TGFb*^50,51^. The correlation analysis was performed between two markers *(Talin1* and *Twist1)* and the results showed that there is no significant correlation between cytoplasmic expression of them in these series of CRC patients (p-value = 0.104).

Additionally, Most of the fifteen genes that were obtained from our bioinformatics analysis, have been previously implicated in cancer development, but *Talin1* was selected due to its most important biological roles in migrant cells. Assembly and disassembly of FAK including *Talin* are essential for cell migration; meanwhile, molecular mechanisms details remained poorly understood and must be elucidated^52^. Up and down regulation of FAK protein expression might have a profound effect on signal transduction; for example, FAK expression levels were increased in primary CRC compared with normal mucosa and decreased in liver metastases^53^. In addition, our findings point towards *Talin1* targeting as a promise for the future, and we supposed that EMT and stemness have been extensively investigated in the field of CTC-related research^54^. With regard to metastasis, the identification of molecular markers that robustly define a subset of CTCs which are enriched in CSCs are vital^55^. Thus, an improved understanding of the EMT/CTC/CSC connections may uncover the novel therapeutic targets and underline tumorigenicity mechanisms^56^.

Several studies have previously investigated levels of *Talin1* expression in CRC in various areas such as by western blot analysis, HPLC-Chip/MS analysis, and enzyme-linked immunosorbent assay (ELISA). Nevertheless, no study was found that addressed the expression of *Talin1* either in mRNA or protein levels in CRC tissue. Hence, due to the existence of information gaps regarding the expression of *Talin1* in both mRNA and protein levels, the present study was designed to examine that in a well-characterized series of 77 fresh and 648 FFEP CRC tissue samples. This is also the first report of localization of *Talin1* expression in cytoplasm and ECM in a large number of tumor samples with evaluation of its impact on CRC prognosis. The current results showed that low level mRNA expression of *Talin1* is associated with increases in TNM stages. Furthermore, the results of TMA revealed an adverse association between the cytoplasmic expression levels of *Talin1* and increasing TNM stage and gender, while no association between the ECM expression of *Talin1* and the clinicopathological parameters was found. It was determined that the localization of *Talin* expression depends completely upon its function related to integrins. Moreover, a statistically significant difference was observed in the median level of *Talin1* mRNA and protein expression between different stages. To clarify, the comparison of low stages (I–II) and high stages (III) showed that *Talin*1 mRNA and protein cytoplasmic expression levels are associated with CRC aggressiveness. It is noteworthy that the continuous refinement of the TNM system, T (tumor), N (node), and M (metastasis) staging define the prognosis and patient outcome and play a pivotal role in decision-making for treatment^57,58^. Accumulating data underlines the importance of structuring the management of CRC patient's survival by stages at diagnosis and prognostic stratification^59,60^. In addition, LNI, NI, distant metastasis, tumor recurrence, and age were prognostic variables in univariate analysis that depicted the associations between these parameters and more aggressive tumor behavior. In cytoplasmic *Talin1* expression, only age was a prognosis factor affecting the DSS in multivariate analysis. It was previously shown that age and comorbidities worsened the survival rate of CRC patients^61^. In this study, the results exhibited that low level cytoplasmic expression of *Talin1* compared with ECM expression is related to the degree of malignancy and progression in CRC cases. *Talin1* cytoplasmic expression can be considered more significant in comparison with ECM ones^62,63^.

Previous articles have shown that there are many challengeable reports regarding *Talin1* expression, analysis, and localization in various tissues. The first study related to *Talin* expression showed that it increased^64^, but eight years later the reduced expression of *Talin* in liver metastases compared with matched primary human CRC was reported^65^. Barbazan et al. introduced one molecular pattern in CRC for CTC detection^66,67^. Their research was continued by Insua et al., who included *Talin1* as a gene involved in CTC extravasation during metastatic dissemination in their gene expression panel, which was composed of *GAPDH, VIL1, CLU, TIMP1, TLN1, LOXL3, and ZEB2*. This panel was checked as a prognostic and predictive tool in metastatic CRC blood samples. Patients with higher gene panel expression compared with low ones had reduced PFS and OS rates (*p* = *0.003* and *p* = *0.001*), respectively^65^. According to the current results, this prognostic value seems to be related to *Talin1* expression accompanied by the other markers, which were important in the panel^53^. One recent study revealed that *Talin1* expression was upregulated in CRC, and the proliferation, migration, and invasive ability of the CRC cell line was significantly reduced by its knockdown compared with the control. In the current study, however, it was downregulated in advanced tumor progression^68^. Analysis of *Talin1* using ELISA showed no significant correlation between its preoperative levels in the serum of patients and age and gender; however, a significant correlation between *Talin1* levels and tumor grade, TNM stage, and lymph node metastasis was found^69^.

The results of the Kaplan–Meier curve on protein determined that tumors with low-level expression of the *Talin1* protein tend to have a worse prognosis for DSS. Moreover, CRC patients who expressed a lower level of cytoplasmic *Talin1* had a worse 5-year survival rate for DSS compared with those with high expression. Thus, low expression level of *Talin1* can be considered a worse prognostic factor of DSS in CRC patients. *Talin1* levels could not be used as an independent prognostic factor of DSS. More prolonged follow-up time may increase the prognostic value of *Talin1*. In mRNA expression, the current finding showed that patients with low *Talin1* mRNA expression had shorter DSS compared to patients with high expression of *Talin1,* but the difference was not statistically significant. This might be due to the limited sample size and shorter follow-up period in comparison with FFEP samples, because all fresh tissue samples were collected on 2017.

The current study was similar to other studies conducted on *Talin1* in some malignancies in its importance in cancer development, invasion, migration, and metastasis, and even introduces it as a cancer stem cell (CSCs) marker, because in the current study, *Talin1* was a common marker between CTCs and exosomes^68,70–72^. Therefore, it must be mentioned that *Talin1* can be an extremely helpful factor for a worse prognosis of DSS in CRC patients. Moreover, the use of IHC can verify the Real-Time PCR results and establish *Talin1* status in routine clinical practice.

## Conclusion

The current study is the first to exhibit that low level *Talin1* mRNA expression is associated with more aggressive tumor behavior in CRC patients. Furthermore, we found that decreased cytoplasmic expression of *Talin1* rather than its ECM expression has a clinical significance in CRC cases and is associated with increased invasiveness and poor prognosis risk for DSS in univariate analysis. To sum up, investigation of the expression pattern of *Talin1* in the cytoplasm of tumor biopsies as a predictor and prognostic indicator of cancer progression in CRC patients’ tumor biopsies is useful. Extending the follow-up time might increase the prognostic value of *Talin1* as an independent prognostic factor, but that must be confirmed in further studies.

## Methods

### Data set collection

To find the common molecular marker between CTCs and exosomes, first, GSE31023 gene expression and CTC profiling by array were obtained from the NCBI GEO database (https://www.ncbi.nlm.nih.gov/geo) based on the GPL13497 (Agilent-026652 Whole Human Genome Microarray 4 × 44 K v2) platforms, containing the CTC in CRC patients (n = 6) and healthy subjects (n = 3). The GEO2R online analysis tool to detect the DEGs was applied, using a *p *value < 0.05 and |logFC|≥ 2 as cut-off criteria.

Secondly, the exosome markers were sought in two known exosome-related websites, https://www.exocarta.org and https://microvesicles.org, which were sequentially searched. A search for CRC cells in Vesiclepedia revealed 37 research sets containing 22,375 proteins and 10,813 mRNA. After duplicates were removed, a total of 6150 proteins and 10,813 mRNA were determined. Another 1617 related CRC exosome proteins were extracted from Exocarta, and all of them are summarized in Supplementary Table 1.

Molecular markers are valuable tools in the development of cancer screening and monitoring strategies. GO and GSEA are the common useful methods employed in this study to suggest new markers among the CTCs and exosomes in CRC samples. GO is useful for annotating genes and identifying characteristic biological attributes for high-throughput data analysis^73^. The GO project describe the attributes of gene products in the molecular functions, biology processes, and cellular components^74^. Gene set enrichment analysis (GSEA) determines whether a prior defined set of genes shows statistical significance^75,76^. Thus, the initially extracted markers in this article were subjected to STRING (https://string-db.org/) and EnrichR (amp.pharm.mssm.edu/Enrichr/) to obtain a better comprehensive of the significantly related pathway, which is described in Supplementary Table 2.

### Patients’ characteristics and tissue collection

In the current study, 77 CRC fresh tumor tissues and their adjacent normal tissue samples from 2018 to 2019 were collected from university-based hospitals (Firoozgar and Bahman) in Tehran, Iran. Patients who had undergone surgery without receiving any relevant chemotherapy or radiotherapy were included in this study. The specimens were embedded in RNA later solution, transferred to the laboratory, and frozen in liquid nitrogen. Patient information including gender, age, TNM stage, tumor differentiation, and distant metastasis was also recorded. To evaluate the protein expression of *Talin1*, 760 FFPE tissues of CRC taken from 2010 to 2019 were collected from university-based hospitals (Firoozgar, Bahman, Rasool, and Hasheminejad) in Tehran, Iran. Hematoxylin and eosin (H&E) stained slides and archived medical records were retrieved to obtain patients’ clinicopathological characteristics, including age, gender, tumor size (maximum tumor diameter), tumor differentiation, TNM stage, venous invasion (VI), lymph node invasion (LNI), neural invasion (NI), distant metastasis, and tumor recurrence. In addition, 60 adjacent normal tissue samples were used to compare expression levels of the *Talin1* markers in a range of tissue samples. From the date of surgery to the date of death was measured as Disease-specific survival (DSS). The interval between the primary surgery and the last follow-up visit was considered as Progression-free survival (PFS) if the patient showed no evidence of disease, metastasis, recurrence, CRC or disease-related death.

### RNA isolation, reverse transcription, and real-time PCR

Briefly, tissue specimens were lysed and homogenized in the presence of a highly denaturing guanidine-thiocyanate-containing buffer that inactivates RNases. Trizol reagent (Sigma, USA) added to provide appropriate binding conditions were grounded. Then, the total RNA was isolated based on the RNAeasy Mini Kit (Qiagen Cat No. /ID: 74,104) protocol. One μg of RNA was reverse transcripted with PrimeScript 1st Strand complementary DNA Synthesis kit (Takara). Real-time PCR assay was performed on the Qiagen Rotor Gen Q system using the SYBR green Premix Dimer Eraser kit (TaKaRa cat number: RR820Q). The cycling conditions were an initial 30 s denaturation at 95 °C and 40 cycles (5 s at 95 °C, 30 s at 60 °C, and 45 s at 72 °C). The GAPDH gene was set as internal control; *Talin1* expression level was detected in forty pairs of CRC and adjacent normal tissue samples. The primer sequences were as follows: *GAPDH*: 5′-AACTTTGGCATTGTGGAAGG-3′ F and 5′-CACATTGGGGGTAGGAACAC-3′ R. *Talin1*: 5′-.TTGGAGATGCCAGCAAGCGACT-3′ F and 5′-CCAGTTCTGTGGCTGCCTGATT-3′. The expression levels of *Talin1* mRNA were normalized against *GAPDH* levels based on the 2^−ΔΔCt^ approach^77^.

### Construction of tissue microarrays (TMAs)

Colorectal tissue TMAs were prepared as described previously^78^. Briefly, our pathologist (Z.S.) examined the H&E slides to select the representative tumor area in each block. Then, using a precision arraying instrument (Tissue Arrayer Minicore; ALPHELYS, Plaisir, France), selected spots were punched into recipient blocks, and TMA blocks were constructed in three copies. In each TMA block, adjacent normal tissue samples were also included to compare the expression pattern of *Talin1* with tumor tissue specimens^79,80^. Due to technical problems and an insufficient amount of tissue for scoring, some cases were lost during the staining and excluded from the study. Tumor heterogeneity is a major concern during the TMA procedure, and thus, at least two or three cores were evaluated from each sample to achieve better results^81,82^.

### Immunohistochemistry (IHC) staining

The protein expression of *Talin1* was evaluated using our IHC laboratory protocol y^29,83^. Sequential TMA sections at 60 °C for 30 min were dewaxed, in xylenes rehydrated, and then under ethanol treatment graded. 3% H_2_O_2_ (endogenous peroxidase) were applied as blocker for 20 min at room temperature. Antigen was retrieved by autoclaving tissue sections for 10 min in sodium citrate buffer (pH 6.0). Then, all of the slides were blocked (blocker protein, Dako, Denmark) for 20 min. After serial dilution, 1:1000 for anti-*Talin1* antibody was prepared as an optimal dilution for subsequent use. The tissue sections were incubated overnight at 4 °C with the following antibody dilutions: anti *Talin1* antibody (ab71333; Abcam, UK). The next day, after three washes in Tris-buffered saline (TBS), sections were incubated with anti-rabbit/anti-mouse envision IgG–HRPO (EnVision, Dako) as the secondary antibody for 1 h. 3,3′-diaminobenzidine (DAB) (Dako) substrate as a chromogen for treating the TMA slides were used in 3 min at room temperature. Sections were counterstained with hematoxylin (Dako), in alcohol dehydrated, with xylenes cleared and mounted. TBS was replaced for negative controls, and only the secondary antibody was used. Meanwhile, human kidney tissue was used as a positive control for *Talin1* staining.

### Evaluation of immunostaining

Our pathologist (Z.S.) using a semi-quantitative scoring examined the immunostained tissue arrays system in a coded manner. Consensus was achieved by the second investigator (Z.M.) in difficult cases for confirming the scoring.

### Scoring system

In the CRC samples, the *Talin1* marker was expressed at various intensities in the cell cytoplasm and ECM, each of which was analyzed separately. The intensities of *Talin1* staining were scored on a 4-point scale, ranging from negative to strong (zero = negative, one = weak, two = moderate, and three = strong). The percentages of positive tumor cells varied between zero and 100. Histochemical score (H-score) as an overall score was achieved by multiplying the intensity of staining by the percentage of positive cells; then, a final score of 0 to 300 was given to each core. In the current study, the mean H-scores were classified into three groups: 1. low expression (0–100), 2. moderate expression (101–200), and 3. high expression (201–300).

### Statistical analysis

SPSS software version 22.0 (IBM Corp, USA) was utilized to analyze the data. Categorical data was reported by N (percentage) and quantitative data as mean (SD) and median (Q1, Q3). Pearson’s χ2 and Spearman’s correlation tests were used to analyze the significance of associations and correlations between *Talin1* expression and clinicopathological parameters. Kruskal–Wallis and Mann–Whitney *U* tests were applied for pairwise comparisons between groups. DSS and PFS curves were drawn using the Kaplan–Meier method, and the log-rank test was used to compare the estimated curves between groups with 95% confidence intervals (CI). To clarify that which variables affected DSS or PFS, the Cox proportional hazards regression model was applied. In all parts, a *p-*value of < 0.05 was considered statistically significant. As noted, in the first step, all quantified data was replicated an average of three times.

### Ethical approval

The Research Ethics Committee of Iran University of Medical Sciences issued IR.IUMS.REC 1395.9221513203 for this study. All procedures, including obtaining informed consent from each human participant before surgery, were in accordance with the above-mentioned ethical standards.

## Supplementary information

Supplementary Information 1.

Supplementary Information 2.
