# Supplementary material for: Low expression of Talin1 is associated with advanced pathological features in colorectal cancer patients
Source: Sci Rep. 2020 Oct 20;10:17786. doi: 10.1038/s41598-020-74810-6 (PMC7576823; doi:10.1038/s41598-020-74810-6)
Supplement: Supplementary file 2 — Supplementary Information 2. [file 41598_2020_74810_MOESM2_ESM.docx]

| **Supplementary Table 2,** Gene Ontologies analysis list | | | |
| --- | --- | --- | --- |
| ID | GO Term | **Gene list** | **P-value** |
| **cellular components** | | | |
| GO:0005925 | focal adhesion | RPS4X;AHNAK;TLN1;RHOB | 1.15E-04 |
| GO:0015935 | small ribosomal subunit | RPS4X;RPSA | 7.08E-04 |
| GO:0022627 | cytosolic small ribosomal subunit | RPS4X;RPSA | 6.05E-04 |
| GO:0022626 | cytosolic ribosome | RPS4X;RPSA | 0.003797763 |
| GO:0044445 | cytosolic part | RPS4X;RPSA | 0.006161458 |
| GO:0030686 | 90S preribosome | RPSA | 0.011937074 |
| GO:0005844 | polysome | RPS4X | 0.046237826 |
| **Biological processes** | | | |
| GO:0006614 | SRP-dependent cotranslational protein targeting to membrane | RPS4X;RPSA | 0.001979927 |
| GO:0006613 | cotranslational protein targeting to membrane | RPS4X;RPSA | 0.002159214 |
| GO:0045047 | protein targeting to ER | RPS4X;RPSA | 0.002345937 |
| GO:0019080 | viral gene expression | RPS4X;RPSA | 0.003003655 |
| GO:0000184 | nuclear-transcribed mRNA catabolic process, nonsense-mediated decay | RPS4X;RPSA | 0.003111689 |
| GO:0019083 | viral transcription | RPS4X;RPSA | 0.003166385 |
| GO:0003159 | morphogenesis of an endothelium | RHOB | 0.004492068 |
| GO:0071635 | negative regulation of transforming growth factor beta production | HSP90AB1 | 0.004492068 |
| GO:0051029 | rRNA transport | RPSA | 0.004492068 |
| GO:0044267 | cellular protein metabolic process | RPS4X;RPSA;TGFBI | 0.005159138 |
| GO:0000447 | endonucleolytic cleavage in ITS1 to separate SSU-rRNA from 5.8S rRNA and LSU-rRNA from tricistronic rRNA transcript (SSU-rRNA, 5.8S rRNA, LSU-rRNA) | RPSA | 0.005238917 |
| GO:1901029 | negative regulation of mitochondrial outer membrane permeabilization involved in apoptotic signaling pathway | SLC25A5 | 0.005238917 |
| GO:0010988 | regulation of low-density lipoprotein particle clearance | CNPY2 | 0.005238917 |
| GO:0002315 | marginal zone B cell differentiation | LFNG | 0.005238917 |
| GO:0071428 | rRNA-containing ribonucleoprotein complex export from nucleus | RPSA | 0.005238917 |
| GO:0045714 | regulation of low-density lipoprotein particle receptor biosynthetic process | CNPY2 | 0.005238917 |
| GO:0022618 | ribonucleoprotein complex assembly | HSP90AB1;RPSA | 0.005938116 |
| GO:2000010 | positive regulation of protein localization to cell surface | HSP90AB1 | 0.005985243 |
| GO:0031328 | positive regulation of cellular biosynthetic process | RPS4X;HSP90AB1 | 0.006161458 |
| GO:0048522 | positive regulation of cellular process | RPS4X;HSP90AB1;SLC25A5 | 0.006264033 |
| GO:0019062 | virion attachment to host cell | HSP90AB1 | 0.006731046 |
| GO:0002313 | mature B cell differentiation involved in immune response | LFNG | 0.006731046 |
| GO:0007016 | cytoskeletal anchoring at plasma membrane | TLN1 | 0.006731046 |
| GO:0000956 | nuclear-transcribed mRNA catabolic process | RPS4X;RPSA | 0.007335155 |
| GO:0043043 | peptide biosynthetic process | RPS4X;RPSA | 0.007335155 |
| GO:0045992 | negative regulation of embryonic development | LFNG | 0.007476328 |
| GO:0061154 | endothelial tube morphogenesis | RHOB | 0.007476328 |
| GO:0010870 | positive regulation of receptor biosynthetic process | CNPY2 | 0.008221088 |
| GO:0000479 | endonucleolytic cleavage of tricistronic rRNA transcript (SSU-rRNA, 5.8S rRNA, LSU-rRNA) | RPSA | 0.008221088 |
| GO:0006048 | UDP-N-acetylglucosamine biosynthetic process | GNPNAT1 | 0.008221088 |
| GO:0031125 | rRNA 3'-end processing | RPSA | 0.008965327 |
| GO:0046349 | amino sugar biosynthetic process | GNPNAT1 | 0.008965327 |
| GO:0006047 | UDP-N-acetylglucosamine metabolic process | GNPNAT1 | 0.008965327 |
| GO:0016072 | rRNA metabolic process | RPS4X;RPSA | 0.009590008 |
| GO:0006364 | rRNA processing | RPS4X;RPSA | 0.009774807 |
| GO:0060330 | regulation of response to interferon-gamma | HSP90AB1 | 0.010452241 |
| GO:0030950 | establishment or maintenance of actin cytoskeleton polarity | RHOB | 0.010452241 |
| GO:0042744 | hydrogen peroxide catabolic process | PRDX1 | 0.010452241 |
| GO:1903320 | regulation of protein modification by small protein conjugation or removal | HSP90AB1 | 0.010452241 |
| GO:0036066 | protein O-linked fucosylation | LFNG | 0.011194917 |
| GO:0032516 | positive regulation of phosphoprotein phosphatase activity | HSP90AB1 | 0.011194917 |
| GO:0016032 | viral process | RPS4X;RPSA | 0.01150938 |
| GO:0042254 | ribosome biogenesis | RPS4X;RPSA | 0.012115774 |
| GO:0034470 | ncRNA processing | RPS4X;RPSA | 0.012218194 |
| GO:0051131 | chaperone-mediated protein complex assembly | HSP90AB1 | 0.01267871 |
| GO:0032387 | negative regulation of intracellular transport | SLC25A5 | 0.01267871 |
| GO:1901028 | regulation of mitochondrial outer membrane permeabilization involved in apoptotic signaling pathway | SLC25A5 | 0.01267871 |
| GO:0006412 | translation | RPS4X;RPSA | 0.012736067 |
| GO:0030511 | positive regulation of transforming growth factor beta receptor signaling pathway | HSP90AB1 | 0.014160425 |
| GO:0000466 | maturation of 5.8S rRNA from tricistronic rRNA transcript (SSU-rRNA, 5.8S rRNA, LSU-rRNA) | RPSA | 0.014160425 |
| GO:0000028 | ribosomal small subunit assembly | RPSA | 0.014160425 |
| GO:1903846 | positive regulation of cellular response to transforming growth factor beta stimulus | HSP90AB1 | 0.014160425 |
| GO:0009226 | nucleotide-sugar biosynthetic process | GNPNAT1 | 0.014160425 |
| GO:0042743 | hydrogen peroxide metabolic process | PRDX1 | 0.014900504 |
| GO:0007004 | telomere maintenance via telomerase | HSP90AB1 | 0.014900504 |
| GO:0071157 | negative regulation of cell cycle arrest | HSP90AB1 | 0.015640064 |
| GO:0006278 | RNA-dependent DNA biosynthetic process | HSP90AB1 | 0.015640064 |
| GO:0010922 | positive regulation of phosphatase activity | HSP90AB1 | 0.016379107 |
| GO:0060334 | regulation of interferon-gamma-mediated signaling pathway | HSP90AB1 | 0.016379107 |
| GO:0032435 | negative regulation of proteasomal ubiquitin-dependent protein catabolic process | HSP90AB1 | 0.017855639 |
| GO:1901385 | regulation of voltage-gated calcium channel activity | AHNAK | 0.018593129 |
| GO:1904407 | positive regulation of nitric oxide metabolic process | HSP90AB1 | 0.018593129 |
| GO:0045429 | positive regulation of nitric oxide biosynthetic process | HSP90AB1 | 0.018593129 |
| GO:1901019 | regulation of calcium ion transmembrane transporter activity | AHNAK | 0.020066558 |
| GO:0010833 | telomere maintenance via telomere lengthening | HSP90AB1 | 0.020066558 |
| GO:2000008 | regulation of protein localization to cell surface | HSP90AB1 | 0.020066558 |
| GO:0070301 | cellular response to hydrogen peroxide | RHOB | 0.020802499 |
| GO:1901799 | negative regulation of proteasomal protein catabolic process | HSP90AB1 | 0.020802499 |
| GO:0045995 | regulation of embryonic development | LFNG | 0.021537924 |
| GO:0045746 | negative regulation of Notch signaling pathway | LFNG | 0.021537924 |
| GO:1903428 | positive regulation of reactive oxygen species biosynthetic process | HSP90AB1 | 0.022272833 |
| GO:0035307 | positive regulation of protein dephosphorylation | HSP90AB1 | 0.023007227 |
| GO:0045428 | regulation of nitric oxide biosynthetic process | HSP90AB1 | 0.023741106 |
| GO:0048813 | dendrite morphogenesis | FARP1 | 0.023741106 |
| GO:0070527 | platelet aggregation | TLN1 | 0.024474471 |
| GO:0010823 | negative regulation of mitochondrion organization | SLC25A5 | 0.024474471 |
| GO:0060562 | epithelial tube morphogenesis | RHOB | 0.025207322 |
| GO:0000462 | maturation of SSU-rRNA from tricistronic rRNA transcript (SSU-rRNA, 5.8S rRNA, LSU-rRNA) | RPSA | 0.025939659 |
| GO:0043666 | regulation of phosphoprotein phosphatase activity | HSP90AB1 | 0.025939659 |
| GO:0061572 | actin filament bundle organization | RHOB | 0.025939659 |
| GO:0051973 | positive regulation of telomerase activity | HSP90AB1 | 0.025939659 |
| GO:0030101 | natural killer cell activation | PRDX1 | 0.026671482 |
| GO:0051017 | actin filament bundle assembly | RHOB | 0.027402792 |
| GO:0034109 | homotypic cell-cell adhesion | TLN1 | 0.02813359 |
| GO:0030217 | T cell differentiation | LFNG | 0.02813359 |
| GO:0001895 | retina homeostasis | PRDX1 | 0.02813359 |
| GO:0051252 | regulation of RNA metabolic process | AHNAK | 0.028863874 |
| GO:0060338 | regulation of type I interferon-mediated signaling pathway | HSP90AB1 | 0.028863874 |
| GO:0034645 | cellular macromolecule biosynthetic process | RPS4X;RPSA | 0.030116802 |
| GO:0071478 | cellular response to radiation | RHOB | 0.031051657 |
| GO:0051972 | regulation of telomerase activity | HSP90AB1 | 0.031051657 |
| GO:0042542 | response to hydrogen peroxide | RHOB | 0.031779896 |
| GO:0006986 | response to unfolded protein | HSP90AB1 | 0.031779896 |
| GO:0042274 | ribosomal small subunit biogenesis | RPSA | 0.032507623 |
| GO:0071479 | cellular response to ionizing radiation | RHOB | 0.033961547 |
| GO:0008333 | endosome to lysosome transport | RHOB | 0.035413431 |
| GO:0071156 | regulation of cell cycle arrest | HSP90AB1 | 0.035413431 |
| GO:0010467 | gene expression | RPS4X;RPSA | 0.037073635 |
| GO:0008284 | positive regulation of cell proliferation | RPS4X;SLC25A5 | 0.039238998 |
| GO:0043484 | regulation of RNA splicing | AHNAK | 0.040479004 |
| GO:2000573 | positive regulation of DNA biosynthetic process | HSP90AB1 | 0.040479004 |
| GO:0042255 | ribosome assembly | RPSA | 0.041200628 |
| GO:0036498 | IRE1-mediated unfolded protein response | TLN1 | 0.041921744 |
| GO:0000281 | mitotic cytokinesis | RHOB | 0.043362459 |
| GO:0034329 | cell junction assembly | TLN1 | 0.043362459 |
| GO:0034250 | positive regulation of cellular amide metabolic process | RPS4X | 0.044801152 |
| GO:0032970 | regulation of actin filament-based process | RHOB | 0.045519741 |
| GO:0007416 | synapse assembly | FARP1 | 0.046237826 |
| GO:0035305 | negative regulation of dephosphorylation | FARP1 | 0.046237826 |
| GO:0007162 | negative regulation of cell adhesion | TGFBI | 0.047672482 |
| GO:0034614 | cellular response to reactive oxygen species | RHOB | 0.047672482 |
| GO:2001234 | negative regulation of apoptotic signaling pathway | SLC25A5 | 0.047672482 |
| GO:1903829 | positive regulation of cellular protein localization | HSP90AB1 | 0.048389055 |
| GO:0010923 | negative regulation of phosphatase activity | FARP1 | 0.049820691 |
| GO:0045088 | regulation of innate immune response | HSP90AB1 | 0.050535755 |
| GO:0010594 | regulation of endothelial cell migration | RHOB | 0.050535755 |
| **Molecular function** | | | |
| GO:0045296 | cadherin binding | HSP90AB1;AHNAK;PRDX1;TLN1 | 7.01E-05 |
| GO:0003723 | RNA binding | RPS4X;HSP90AB1;AHNAK;PRDX1;RPSA;SLC25A5 | 3.19E-04 |
| GO:1990226 | histone methyltransferase binding | HSP90AB1 | 0.004492068 |
| GO:0005345 | purine nucleobase transmembrane transporter activity | SLC25A5 | 0.004492068 |
| GO:0005347 | ATP transmembrane transporter activity | SLC25A5 | 0.006731046 |
| GO:0017166 | vinculin binding | TLN1 | 0.007476328 |
| GO:0015217 | ADP transmembrane transporter activity | SLC25A5 | 0.007476328 |
| GO:0030676 | Rac guanyl-nucleotide exchange factor activity | FARP1 | 0.010452241 |
| GO:0070182 | DNA polymerase binding | HSP90AB1 | 0.011937074 |
| GO:0023026 | MHC class II protein complex binding | HSP90AB1 | 0.011937074 |
| GO:0023023 | MHC protein complex binding | HSP90AB1 | 0.014160425 |
| GO:0015301 | anion:anion antiporter activity | SLC25A5 | 0.015640064 |
| GO:0008080 | N-acetyltransferase activity | GNPNAT1 | 0.016379107 |
| GO:0097718 | disordered domain specific binding | HSP90AB1 | 0.017117631 |
| GO:0043022 | ribosome binding | RPSA | 0.02813359 |
| GO:0035639 | purine ribonucleoside triphosphate binding | HSP90AB1;RHOB | 0.034636495 |
| GO:0008375 | acetylglucosaminyltransferase activity | LFNG | 0.036863279 |
| GO:0019900 | kinase binding | HSP90AB1;RHOB | 0.038233536 |
| GO:0005518 | collagen binding | TGFBI | 0.038311092 |
| GO:0005089 | Rho guanyl-nucleotide exchange factor activity | FARP1 | 0.043362459 |
| GO:0003725 | double-stranded RNA binding | HSP90AB1 | 0.043362459 |
| GO:0045296 | cadherin binding | HSP90AB1;AHNAK;PRDX1;TLN1 | 7.01E-05 |
| GO:0003723 | RNA binding | RPS4X;HSP90AB1;AHNAK;PRDX1;RPSA;SLC25A5 | 3.19E-04 |
| GO:1990226 | histone methyltransferase binding | HSP90AB1 | 0.004492068 |
| GO:0005345 | purine nucleobase transmembrane transporter activity | SLC25A5 | 0.004492068 |
| GO:0005347 | ATP transmembrane transporter activity | SLC25A5 | 0.006731046 |
| GO:0017166 | vinculin binding | TLN1 | 0.007476328 |
| GO:0015217 | ADP transmembrane transporter activity | SLC25A5 | 0.007476328 |
| GO:0030676 | Rac guanyl-nucleotide exchange factor activity | FARP1 | 0.010452241 |
| GO:0070182 | DNA polymerase binding | HSP90AB1 | 0.011937074 |
| GO:0023026 | MHC class II protein complex binding | HSP90AB1 | 0.011937074 |
| GO:0023023 | MHC protein complex binding | HSP90AB1 | 0.014160425 |
| GO:0015301 | anion:anion antiporter activity | SLC25A5 | 0.015640064 |
| GO:0008080 | N-acetyltransferase activity | GNPNAT1 | 0.016379107 |
| GO:0097718 | disordered domain specific binding | HSP90AB1 | 0.017117631 |
| GO:0043022 | ribosome binding | RPSA | 0.02813359 |
| GO:0035639 | purine ribonucleoside triphosphate binding | HSP90AB1;RHOB | 0.034636495 |
| GO:0008375 | acetylglucosaminyltransferase activity | LFNG | 0.036863279 |
| GO:0019900 | kinase binding | HSP90AB1;RHOB | 0.038233536 |
| GO:0005518 | collagen binding | TGFBI | 0.038311092 |
| GO:0005089 | Rho guanyl-nucleotide exchange factor activity | FARP1 | 0.043362459 |
| GO:0003725 | double-stranded RNA binding | HSP90AB1 | 0.043362459 |
| **TLN1,** Talin-1  **TMEM51,** Transmembrane protein 51  **GNPNAT1,** Glucosamine 6-phosphate N-acetyltransferase  **RPS4X,** Ribosomal Protein S4 X-Linked  **MARCKSL1,** MARCKS-related protein  **FARP1,** FERM, ARH/RhoGEF And Pleckstrin Domain Protein 1  **RHOB,** Rho-related GTP-binding protein  **HSP 90AB1,** Heat Shock Protein 90 Alpha Family Class B Member 1  **LFNG,** Beta-1,3-N-acetylglucosaminyltransferase lunatic fringe  **AHNAK,** Neuroblast differentiation-associated protein  **RPSA,** Ribosomal Protein SA  **CNPY2,** Protein canopy homolog 2  **PRDX1,** Peroxiredoxin-1  **SLC25A5,** Solute Carrier Family 25 Member 5  **TGFBI,** Transforming growth factor-beta-induced protein ig-h3 | | | |
